# Draft genome sequence of *Brevibacterium linens* MA5, an isolate from Danbo cheese that carries the bacteriocin gene cluster

**DOI:** 10.1128/mra.00963-25

**Published:** 2025-10-10

**Authors:** Shuangqing Zhao, Belay Tilahun Tadesse, Liuyan Gu, Christian Solem

**Affiliations:** 1 National Food Institute, Technical University of Denmark 5205, Lyngby, Denmark; Fluxus Inc., Sunnyvale, California, USA

**Keywords:** whole genome, *Brevibacterium linens*, linocin M18

## Abstract

*Brevibacterium linens* MA5 was isolated from the rind of a Danish Danbo cheese. Its 3.7 Mb draft genome comprises 3,369 CDS, 8 rRNAs, 47 tRNAs, and 3 ncRNAs. Genomic comparison confirmed borderline species-level identity to *B. linens* ATCC 19391. Genome mining identified a putative bacteriocin gene cluster.

## ANNOUNCEMENT

*Brevibacterium linens* produces proteolytic enzymes that hydrolyze casein into peptides and amino acids, contributing to curd softening and flavor development during smear cheese ripening (1).

Here, we present the draft genome of *B. linens* MA5, which was isolated from the rind of Danbo cheese (ripened for 2 weeks) at Mammen Dairies (Mammen Mejerierne A/S, Bjerringbro, Denmark, 56.3796°N, 9.6403°E). Microorganisms were washed off cheese rind samples using sterile physiological saline and inoculated onto Brain Heart Infusion (BHI) agar. Single colonies of *B. linens* MA5 were obtained after incubation at 30°C for 24 h and purified by repeated streaking. DNA was extracted from an early stationary-phase culture grown in 50 mL BHI broth (24 h, 30°C, 200 rpm). Total DNA was isolated from 1 mL *B. linens* MA5 culture using the DNeasy blood and tissue kit (Qiagen, Germany). Genomic DNA quality and quantity were assessed using an Agilent 5400 system (Agilent Technologies, CA, USA). The DNA concentration was 10.38 ng/µL and passed the QC evaluation. Genomic DNA was randomly fragmented and processed for end-repair, A-tailing, and ligation of Illumina adapters using the Illumina DNA Prep library preparation kit (Illumina, CA, USA). Adapter-ligated fragments were size-selected, PCR-amplified, and purified with AMPure XP beads (Beckman Coulter, MA, USA). Library quality was assessed using a fragment analyzer (Agilent, CA, USA), and libraries were quantified with Qubit (Thermo Fisher Scientific, MA, USA) and qPCR before sequencing on the Illumina NovaSeq X Plus platform (paired-end 150 bp) at Novogene (Munich, Germany).

The read length was 150 bp, and the number of reads in total was 11.9 million with the sequencing depth 35.6×. The raw reads were subsequently quality checked by fastp 1.0 (2), whereafter adapter sequences and low-quality sequences were trimmed from both ends. This process resulted in 11.7 million sequences, with an average length of 149 bp. The genome was assembled using Unicycler v.0.5.0 (3). The completeness and contamination of genome bins were assessed using lineage-specific marker sets using checkM V1.2.4 (4), and the genome was 94.18% complete with 3.35% contamination and strain heterogeneity. The assembly contained 114 contigs, with a total length of 3.7 Mb and an N50 of 106.6 kb. The assembled genomes were annotated using NCBI PGAP 6.10 (5). The GC content was 64.5%, with 3,369 CDS, 8 rRNAs, 47 tRNAs, and 3 ncRNAs. Genome similarity analysis showed high relatedness to *Brevibacterium linens*, with 70.8% dDDH as determined using TYGS (6) and 93.99% average nucleotide identity, as determined using fastANI v1.34 (7). Genome mining of *B. linens* MA5 using antiSMASH 8.0 (8) and BAGEL4 (9) identified a putative bacteriocin gene cluster encoding linocin M18 (10). All software was used with default settings.

## Data Availability

The genome sequence of B. linens strain MA5 has been stored at NCBI under the Bioproject (accession number: PRJNA1304246). Raw data have been deposited in the Sequence Read Archive (SRA) under accession numbers (SRR34941584). Submitted GenBank assembled genome for B. linens MA5 under accession number (JBQGQF000000000).
